# Mesenchymal Phenotype of CTC-Enriched Blood Fraction and Lymph Node Metastasis Formation Potential

**DOI:** 10.1371/journal.pone.0093901

**Published:** 2014-04-07

**Authors:** Aleksandra Markiewicz, Magdalena Książkiewicz, Marzena Wełnicka-Jaśkiewicz, Barbara Seroczyńska, Jarosław Skokowski, Jolanta Szade, Anna J. Żaczek

**Affiliations:** 1 Department of Medical Biotechnology, Intercollegiate Faculty of Biotechnology, University of Gdańsk and Medical University of Gdańsk, Gdańsk, Poland; 2 Postgraduate School of Molecular Medicine, Medical University of Warsaw, Warsaw, Poland; 3 Department of Oncology and Radiotherapy, Medical University of Gdańsk, Gdańsk, Poland; 4 Bank of Frozen Tissues and Genetic Specimens, Department of Medical Laboratory Diagnostics, Medical University of Gdańsk, Gdańsk, Poland; 5 Department of Surgical Oncology, Medical University of Gdańsk, Gdańsk, Poland; 6 Department of Pathomorphology, Medical University of Gdańsk, Gdańsk, Poland; National University of Ireland Galway, Ireland

## Abstract

**Introduction:**

Circulating tumor cells (CTCs) that present mesenchymal phenotypes can escape standard methods of isolation, thus limiting possibilities for their characterization. Whereas mesenchymal CTCs are considered to be more malignant than epithelial CTCs, factors responsible for this aggressiveness have not been thoroughly defined. This study analyzed the molecular profile related to metastasis formation potential of CTC-enriched blood fractions obtained by marker unbiased isolation from breast cancer patients without (N−) and with lymph nodes metastases (N+).

**Materials and Methods:**

Blood samples drawn from 117 patients with early-stage breast cancer were enriched for CTCs using density gradient centrifugation and negative selection with anti-CD45 covered magnetic particles. In the resulting CTC-enriched blood fractions, expression of *CK19, MGB1, VIM, TWIST1, SNAIL, SLUG, HER2, CXCR4* and *uPAR* was analyzed with qPCR. Results were correlated with patients' clinicopathological data.

**Results:**

CTCs (defined as expression of either *CK19, MGB1* or *HER2*) were detected in 41% (20/49) of N− and 69% (34/49) of N+ patients (*P* = 0.004). CTC-enriched blood fractions of N+ patients were more frequently *VIM* (*P* = 0.02), *SNAIL* (*P* = 0.059) and *uPAR*-positive (*P* = 0.03). Positive *VIM, CXCR4* and *uPAR* status correlated with >3 lymph nodes involved (*P* = 0.003, *P* = 0.01 and *P* = 0.045, respectively). In the multivariate logistic regression *MGB1* and *VIM*-positivity were independently related to lymph node involvement with corresponding overall risk of 3.2 and 4.2. Moreover, mesenchymal CTC-enriched blood fractions (*CK19−/VIM+* and *MGB1+* or *HER2+*) had 4.88 and 7.85-times elevated expression of *CXCR4* and *uPAR*, respectively, compared with epithelial CTC-enriched blood fractions (*CK19+/VIM−* and *MGB1+* or *HER2+*).

**Conclusions:**

Tumors of N+ patients have superior CTC-seeding and metastatic potential compared with N- patients. These differences can be attributed to *VIM, uPAR* and *CXCR4* expression, which endow tumor cells with particularly malignant phenotypes.

## Introduction

Presence of circulating tumor cells (CTCs) in blood of patients with epithelial cancer was first demonstrated by Ashworth in 1869 [1]. As techniques were developed to capture, enumerate and characterize circulating and disseminated tumor cells, significant progress was made in understanding metastatic processes [2], [3], [4], [5]. The number of CTCs detected in blood samples carry prognostic information in early [6], [7] and metastatic breast cancer [8], [9]. Also, CTCs detected via PCR-based methods (without the possibility of cell enumeration) have been associated with poor prognosis in a number of studies [10], [11], described in a recent meta-analysis [12].

As CTCs originate from the epithelium, use of epithelial markers (eg, cytokeratins, EpCAM) for their detection seems reasonable. Cytokeratin 19 (CK19) is a cytoskeletal protein of epithelial cells (both normal and cancerous) and is widely used for detection of CTCs [13], [14], [15] and DTCs [15], [16], [17], [18]. However, discovery of epithelial–mesenchymal transition (EMT) in cancer educes a reconsideration of CTCs as having exclusively epithelial phenotype [2], [19], [20], [21]. Moreover, the role of EMT in cancer implies that detection methods that rely solely on epithelial markers (or other markers downregulated during EMT) are likely to miss the most aggressive fraction of CTCs [2], [19], [22]. Thus, to increase sensitivity it is suggested to include additional mammary transcripts, like mammaglobin 1 (MGB1), which was shown to be a useful marker for detecting disseminated breast cancer cells in blood [23], [24], [25], bone marrow [26], [27] and lymph nodes [28], [29], [30]. Additionally to *CK19* and *MGB1*, detection of *HER2* transcripts, which is frequently overexpressed in breast cancers, strengthened prognostic value of the RT-qPCR based CTCs detecting assay [23].

Activation of EMT is linked to motility, stem cell characteristics, enhanced chemo- and radiotherapy resistance [19], [31], [32], [33]. The fraction of CTCs with a mesenchymal phenotype reportedly reaches almost 100% in the blood of some breast cancer patients [3]. Moreover, in some patients, disease progression during treatment was related to increased number of mesenchymal CTCs compared with their pre-treatment state [2]. The ability of tumor cells to metastasize can be modified by expression of various invasion and metastasis-related factors. Plasminogen activator, urokinase receptor (uPAR) constituting a part of uPA-PAI extracellular matrix degradation system might facilitating tumor cells invasion, migration and growth [34], [35]. *uPAR* was also shown to be amplified together with *HER2* in breast cancer CTCs [36] and decreased expression of uPAR related to tumor cell dormancy [35]. Yet another protein, CXCR4 chemokine receptor, apart from being involved in metastases formation and migration of cancer cells to specific organs [37], [38] is functionally linked with HER2 signalling and malignant progression. CXCR4 expression is enhanced by HER2, which can together act in multiple steps of metastatic cascade [39].

Inherent increased malignancy of mesenchymal CTCs could also contribute to higher metastatic potential, which in early-stage breast cancer could be measured by lymph-node involvement. We have hypothesized that CTCs isolated from lymph node-negative (N−) and -positive (N+) patients could differ in expression of malignancy-associated genes. We therefore used a marker-unbiased CTC-enrichment method that enriches both epithelial and mesenchymal CTCs, in which we measured expression of mammary epithelial transcripts (*CK19, MGB1*), EMT-related factors (*VIM, TWIST1, SNAIL, SLUG*) and invasion- and metastasis- related genes (*HER2, CXCR4, uPAR*).

## Methods

### Patients

The study included 117 breast cancer patients, stages I–III, treated in the Medical University Hospital in Gdansk between April 2011 and May 2013. Tumor stage and node positivity were defined according to AJCC cancer staging manual version 7. Micrometastases were considered and there was one case in examined group identified, which we classified as N+ patient. There were no patients with isolated tumor cells in the N- group. Cancers were graded according to modified Bloom and Richardson system based on semi-quantitative method for assessing histological grade in breast tumours [40]. Median age of the patients was 61 years (28–89 years) (**Table 1**). Inclusion criteria were primary operable breast cancer confirmed by histological examination, and signed consent form. Peripheral blood samples (5–10 mL) were drawn to EDTA-coated tubes before tumor excision and therapy initiation. The first few milliliters of blood were discarded to minimize possibility of keratinocyte contamination. Samples were stored at 4 °C until analysis, but no longer than 24 h. Blood samples from 17 healthy women (median age 36 years; range 20–73 years) and three women with benign breast disease (ductal carcinoma *in situ* – DCIS, median age 65 years; range 46–71) were similarly drawn and processed.

**Table 1:** Patients' characteristics (N = 117).

Variable	Number of cases (%)	
**Age – median (range)**	61	(28–89)
**T stage**		
T1	50	(43)
T2	58	(50)
T3	5	(4)
T4	3	(3)
**Missing data**	1	(1)
N stage		
N-	60	(51)
N+	57	(49)
**Grade**		
G1	15	(13)
G2	63	(54)
G3	39	(33)
**HER2 status**		
Negative	88	(75)
Positive	26	(22)
Missing data	3	(3)
**ER status**		
Negative	23	(20)
Positive	94	(80)
**PR status**		
Negative	29	(25)
Positive	88	(75)
**Histological type**		
Ductal	88	(75)
Lobular	16	(14)
Other	12	(10)
Missing data	1	(1)

Median follow-up time was 1.5 years (0.2 to 2.2 years). Including the last follow-up data six deaths were observed, which is insufficient for performing survival analysis; however, follow-up data continue to be collected.

Hormone receptors status (ER and PgR) was evaluated using classical Allred scoring method with 3 being a cut-off point for positive result. Standard criteria for evaluating HER2 positivity were applied, being 3+ score in immunohistochemistry or positive result in fluorescence in situ hybridization (FISH), as previously described [41].

### Ethics statement

The study was approved by the local Ethical Committee of the Medical University of Gdansk and the manuscript was prepared according to the REMARK criteria [42].

### CTCs isolation/enrichment

**Figure 1:**
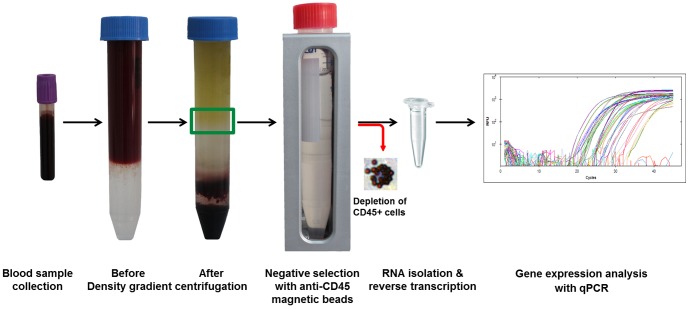
Schematic representation of blood samples analysis process. Collected blood sample was layered on density gradient. After centrifugation fraction containing tumor cells (marked by green box) was collected and subjected to negative selection. After CD45-depletetion, CTC-enriched blood sample was subjected to RNA isolation, reverse transcription and gene expression analysis with qPCR.

For the immunofluorescence experiment, blood samples were first centrifuged at 200 g for 20 min at 20 °C to remove excessive platelets that hampered cell adhesion to polylysine slides after CTC-enrichment. The top serum layer, which contained platelets, was collected and discarded; the remaining fraction was then processed with the full blood sample intended for RNA isolation. Blood samples, or platelet-removed fractions (5 mL), were subjected to CTC-enrichment as described before [43] (**Figure 1**). Briefly, phosphate buffered saline (PBS) diluted blood sample was transferred into a 15 ml tube containing two-layer density gradient (upper and lower gradient) and centrifuged to separate tumor cells containing fraction from erythrocytes and blood cells. The tumor cell containing fraction was collected and subjected to further depletion of CD45-positive cells with anti-CD45-covered magnetic particles (CD45 Dynabeads, Invitrogen). After depletion, the obtained cells pellets were used in the RNA isolation procedure, or if cells were to be visualized by immunofluorescence, pellets were suspended in 1 mL PBS buffer and spun down on 2–4 polylysine-coated glass slides in Rotofix 32A (Hettich).

To evaluate cell recovery rates in the developed CTC-enrichment method, a spike-in experiment was carried out twice. On average, 6 and 11 MDA-MB-361 cells (5 and 11 in the first experiment; 6 and 10 cells in the second experiment) were spiked to peripheral blood samples from healthy women, which were then processed as the patients' samples. Four cytospins were prepared from each sample, slides were stained for CK19 and CD45 as in the immunofluorescence experiment and analyzed under Axiovert 200 (Zeiss) microscope.

### RNA isolation and qRT-PCR

RNA was isolated with Trizol reagent (Invitrogen) according to manufacturer's instructions. Up to 10 μl of RNA was used in a reverse transcription reaction (Transcriptor FirstStrand Synthesis Kit, Roche) with random hexamers according to manufacturer's instructions. Presence of inhibitors in the isolated RNA was tested using exogenous RNA molecules (Solaris RNA Spike Control Kit, Thermo Scientific) according to manufacturer's instructions, adding 1 μl of 100× Solaris Spike solution per 1 μg of RNA. cDNA was diluted and 10 ng of cDNA (4 μL) was used in a single 20-μL qPCR reaction. Universal PCR Mastermix (with UNG AmpErase) and TaqMan Gene Expression Assays were used to measure expression of nine genes of interest: *TWIST1* (Hs00361186_m1; UniGene Hs.644998), *SNAIL* (also known as *SNAI1*, Hs00195591_m1; UniGene Hs.48029), *SLUG* (also known as *SNAI2*, Hs00950344_m1; UniGene Hs.360174), *CK19* (Hs01051611_gH; UniGene Hs.654568), *MGB1* (Hs00935948_m1; UniGene Hs.46452), *HER2* (Hs99999005_mH; UniGene Hs.446352), *CXCR4* (Hs00237052_m1; UniGene Hs.593413), *uPAR* (Hs00182181_m1; UniGene Hs.466871), *VIM* (Hs00185584_m1; UniGene Hs.455493) and two reference genes *GAPDH* (Hs99999905_m1; UniGene Hs.544577) and *YWHAZ* (Hs03044281_g1; UniGene Hs.492407). Reference genes were chosen for their expression stability as assessed by geNorm according to the provided manual [43]. qPCR reactions were performed in duplicate on 96-well plates in a CFX96 thermal cycler (Bio-Rad). Cycling parameters were as follows: 10 min 95 °C, 45 cycles of 1 min at 60 °C followed by 30 s at 95 °C. For the TaqMan assays detecting genomic DNA (*CK19* and *YWHAZ*), sample-specific controls (10 ng of untranscribed RNA) were included on every plate. Samples were assumed to be genomic DNA-free if differences between control and test samples were ≥5 CT. Gene expression was calculated using a modified ΔΔCt method that corrects for a run-to-run variation [44]. Therefore, every plate included an inter-run calibrator that allowed calculation of calibrator-normalized relative quantities using qBasePLUS software version 2.1 [44]. Gene expression levels were scaled to the minimal expression level of each gene in the patients' samples.

### Immunofluorescence

As PCR-based techniques do not allow for cells visualization, immunofluorescence experiments were carried out for some patient samples to visualize CTCs isolated using the developed method (described in “CTCs isolation/enrichment”). Double-staining experiments were performed for the following proteins combinations: CK19 and CD45, CK19 and MGB1, CXCR4 and CD45, HER2 and CD45, SNAIL and CD45. Primary mouse monoclonal (CK19, CXCR4, SNAIL, HER2) or rabbit polyclonal (CD45, MGB1) antibodies were used (Santa Cruz Biotechnology; Abnova for anti-SNAIL antibody). Cytospins, prepared on polylysine-coated slides as described above, were fixed with cold methanol for 5 minutes and incubated with appropriate pairs of primary antibodies for 1 hour at room temperature. Pairs of mouse monoclonal antibodies and rabbit polyclonal antibodies were used. Slides were then stained for 1 h with corresponding secondary antibodies: Sheep Anti-Mouse DyLight 549 and Goat Anti-Rabbit DyLight 488 (JIR). Cell nuclei in all slides were stained with 4,6-diamidino-2-phenylindole (Sigma). Slides were evaluated using inverted fluorescent microscope Axiovert 200 (Zeiss) and analyzed with AxioVision software (Zeiss).

### Statistical analysis

Statistical analyses were performed with STATISTICA software version 10. Categorical variables were analysed using contingency tables with χ2 statistics or Fisher's exact test where applicable. Spearman rank coefficient was used when continuous variables were correlated; Mann-Whitney test for analyzing continuous variables distribution in two groups. Logistic regression analysis was used to identify the gene predictors of lymph node involvement. Univariate predictors significant with a value of p≤0.10 were entered into a step-wise backwards multivariate model to identify those with independent prognostic information.

Unsupervised hierarchical clustering analysis was performed using GenePattern version 6 software (http://genepattern.broadinstitute.org/) [45]. Spearman's rank correlation was applied for columns and rows distance measure. Pairwise complete-linkage was used as a clustering method. Samples with missing gene expression values were removed from the analysis.

## Results

### CTC-enriched blood samples characterization

For the average 6 and 11 MDA-MB-361 cells added to blood samples, the CTC-enrichment method showed average recovery rate of 54% and 72%, respectively. In every case, 2–4 cells were lost during sample processing. Double staining (staining of CD45 and another protein of interest) revealed CK19+/CD45−, HER2+/CD45−, CXCR4+/CD45− and SNAIL+/CD45− cells isolated from breast cancer patients (**Figure S1**). In CK19/MGB1 double staining, following phenotypes occurred: CK19+/MGB1+, CK19+/MGB1− and CK19−/MGB1+ (**Figure S1**).

Functional RNA was successively isolated from 84% (98/117) of patient samples and 65% (11/17) of controls. None of the control samples was positive for *TWIST1, SNAIL, SLUG, HER2* or *uPAR*; 1 out of 11 showed *CXCR4* expression (relative gene expression level 4.54); and 9 (82%; 9/11) showed *VIM* expression (median relative gene expression level 11.44, range 0–30.67). Expression of *MGB1* and *CK19* was analyzed in a qualitative manner. Patient samples were considered positive when measured relative gene expression level was higher than the highest measured relative expression level in the control samples. **Table 2** presents the number of samples expressing analyzed genes according to this positivity criterion. Fifty-five percent (54/98) of the patient samples with invasive tumors were positive for either *CK19, MGB1* or *HER2*. In total, 26% of the samples expressed at least one EMT marker (*VIM, TWIST1, SNAIL* or *SLUG*). No healthy control expressed CTCs markers, whereas in all three cases of DCIS expression of at least one CTC marker was detected (**Table 2** and **Figure S2**).

**Table 2:** Relative gene expression levels in CTC-enriched blood fractions from breast cancer patients.

	Gene	Number of samples*		% of positive samples	Average expression	SD	Median	Expression minimum	Expression maximum
Breast cancer patients		positive	negative						
	***TWIST1***	2	94	2	0.05	0.39	0	0	3.71
	***SNAIL***	8	88	8	0.32	1.64	0	0	14.38
	***SLUG***	0	92	0	-	-	-	-	-
	***VIM***	19	75	20	19.40	17.70	14.27	0	105.10
	***HER2***	34	61	36	8.33	18.26	0	0	96.50
	***CXCR4***	40	57	41	5.34	7.79	3.34	0	48.42
	***uPAR***	57	40	59	14.65	22.73	4.50	0	118.80
	***CK19***	26	72	27	1056.88	1054	0	0	2585
	***MGB1***	23	71	24	1714.53	1871	0	0	4424
**DCIS**									
	***TWIST1***	0	3	0	-	-	-	-	-
	***SNAIL***	1	2	33	3.01	5.21	0	0	9.02
	***SLUG***	0	3	0	-	-	-	-	-
	***VIM***	0	3	0	20.18	7.17	22.28	12.2	26.07
	***HER2***	2	1	67	7.66	8.76	5.78	0	17.21
	***CXCR4***	1	2	33	7.07	9.06	3.93	0	17.28
	***uPAR***	2	1	67	9.79	8.48	14.49	0	14.88
	***CK19***	1	2	33	17.81	30.85	0	0	53.43
	***MGB1***	3	0	100	973.24	1567.26	17.93	17.93	2782
**Healthy controls** **									
	***VIM***	Not applicable	Not applicable	Not applicable	13	9.40	11.44	0	30.70
	***CXCR4***	Not applicable	Not applicable	Not applicable	0.40	1.40	4.54	0	4.54

*Number of positive and negative samples for a particular gene, percentages of samples classified as marker-positive with the chosen cut-off levels as well as average expression with standard deviation (SD), minimal and maximal measured relative expression level.

**In the healthy control group only genes which expression was found in the samples are shown.

**Figure 2:**
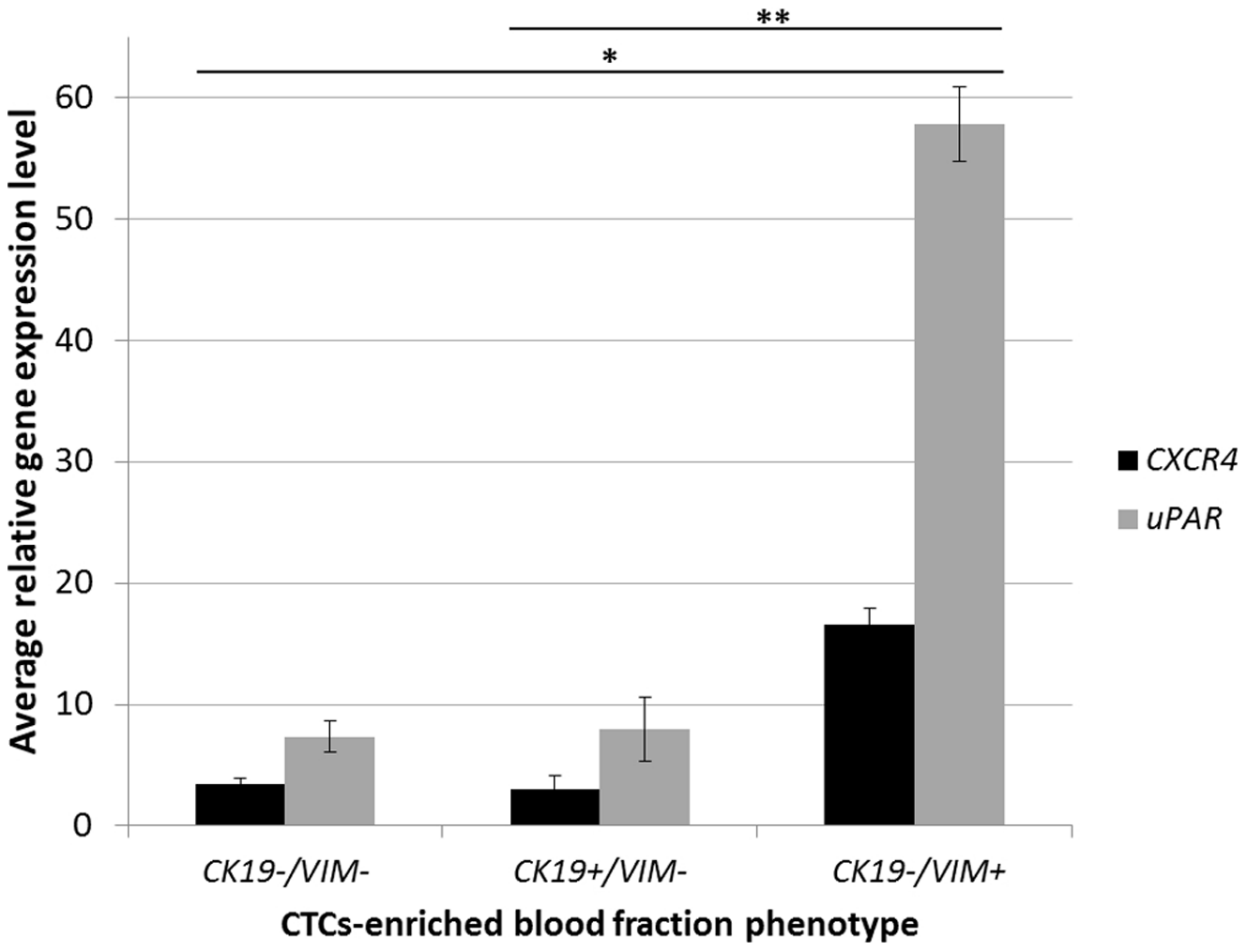
Relative expression level of *CXCR4* and *uPAR* in CTC-enriched blood fractions. CTC-enriched blood fractions positive for either *MGB1* or *HER2* were divided according to *VIM* and *CK19* expression status into three groups (*CK19−/VIM−*; *CK19+/VIM−*; *CK19−/VIM+*). Error bars depict standard error. * - statistically significant difference in *CXCR4* and *uPAR* relative expression level (*P*<0.00001 for both) between *CK19−/VIM−* and *CK19−/VIM+*; ** - statistically significant difference in *CXCR4* (*P* = 0.00002) and *uPAR* (*P* = 0.00004) relative expression level between *CK19+/VIM*− and *CK19−/VIM+*.

CTC-enriched samples expressing at least one of CTCs markers – *MGB1* or *HER2* were divided by *CK19* and *VIM* gene expression status into four groups with positive or negative expression status. Phenotype frequencies were as follow: *CK19+/VIM*− 17% (16/94), *CK19+/VIM+* 1% (1/94), *CK19−/VIM+* 13% (12/94) and *CK19−/VIM−* 69% (65/94). *CK19+/VIM+* sample was excluded from statistical analysis to avoid biased classification to either *CK19+/VIM−* or *CK19−/VIM+* group. Expression of *CXCR4* and *uPAR* was higher in *VIM*-positive fraction (*CK19−/VIM+*) than in the *CK19−/VIM−* CTC-enriched blood fraction (**Figure 2**). In *CK19*−/*VIM+* CTC-enriched blood fractions, average expression of *CXCR4* and *uPAR* was 4.88 and 7.85-fold higher than in *CK19−/VIM−* fraction, respectively (*P* < 0.00001 for both).

### Correlations between gene expression of examined markers

*SNAIL* expression was found in 8% (8/96) of the samples; its expression correlated with positive status of *VIM* (*P* = 0.03), *HER2* (*P* = 0.0002), *CXCR4* (*P* = 0.006) and *uPAR* (*P* = 0.02) (**Table 3**). CTC-enriched *HER2*-positive blood samples were more frequently *VIM*-positive (38% vs. 12%, *P* = 0.004), *SNAIL*-positive (24% vs. 0%, *P* = 0.0002), *CXCR4*-positive (53% vs. 33%, *P* = 0.05) and *uPAR*-positive (100% vs. 34%, *P*<0.00001) than *HER2*-negative samples (**Table 3**). Moreover, 82% (32/39) of CTC-enriched *CXCR4*-positive samples were also *uPAR-*positive (*P* = 0.0001; **Table 3**). Spearman rank correlations between relative expression of the tested genes showing similar gene dependence are presented in **Table S1**.

**Table 3:** Correlations between genes expression status in CTC-enriched blood fractions.

		*TWIST1*			*SNAIL*			*CK19*			*MGB1*			*VIM*			*HER2*			*CXCR4*		
		Neg.	Pos.	*P*	Neg.	Pos.	*P*	Neg.	Pos.	*P*	Neg.	Pos.	*P*	Neg.	Pos.	*P*	Neg.	Pos.	*P*	Neg.	Pos.	*P*
***SNAIL***	**Neg.**	87	1	0.16																		
	**Pos.**	7	1																			
	**Total**	94	2																			
***CK19***	**Neg.**	70	2	1	65	7	0.67															
	**Pos.**	24	0		23	1																
	**Total**	94	2		88	8																
***MGB1***	**Neg.**	70	1	0.43	65	6	1	61	10	**0.00004**												
	**Pos.**	22	1		22	1		10	13													
	**Total**	92	2		87	7		71	23													
***VIM***	**Neg.**	72	1	0.37	70	3	**0.03**	52	23	0.076	55	18	1									
	**Pos.**	18	1		14	4		17	2		15	4										
	**Total**	90	2		85	7		69	25		70	22										
***HER2***	**Neg.**	61	0	0.13	61	0	**0.0002**	48	13	0.24	49	12	0.21	53	7	**0.004**						
	**Pos.**	32	2		26	8		23	11		22	10		20	12							
	**Total**	93	2		87	8		71	24		71	22		73	19							
***CXCR4***	**Neg.**	56	1	1	56	1	**0.006**	40	17	0.42	42	15	0.45	53	3	**0.00001**	41	16	**0.05**			
	**Pos.**	37	1		31	7		31	9		29	7		22	16		20	18				
	**Total**	93	2		87	8		71	26		71	22		75	19		61	34				
***uPAR***	**Neg.**	40	0	0.51	40	0	**0.02**	33	7	0.12	34	6	0.07	34	5	0.13	40	0	**<0.00001**	33	7	**0.0001**
	**Pos.**	53	2		47	8		39	18		37	17		41	14		21	33		24	32	
	**Total**	93	2		87	8		72	25		71	23		75	19		61	33		57	39	

Number of cases classified as positive (Pos.) or negative (Neg.) for a particular marker are shown. Statistically significant *P* values are given in bold.

### Correlation with clinicopathological data

In CTC-enriched blood fractions of N+ patients, at least one of the markers – *CK19, MGB1* or *HER2* – was more frequently (69%, 34/49) detected than in N- patients (41%, 20/49; *P* = 0.004, **Table 4**). Concerning phenotype of CTC-enriched blood fractions, there was no difference between lymph node involvement in patients with epithelial and mesenchymal CTC-enriched blood fraction phenotype (*P* = 0.69), however mesenchymal phenotype was more frequently found in patients with more than 3 lymph nodes involved −50% in comparison to patients with epithelial CTC-enriched blood fraction phenotype −7% (*P* = 0.008, **Table 5**). Moreover, in N+ patients, CTC-enriched blood fractions were more often *MGB1, SNAIL, VIM* and *uPAR*-positive (*P* = 0.03, *P* = 0.059, *P* = 0.02 and *P* = 0.03, respectively) (**Table 5**). Positive status of *CXCR4* was associated with higher T stage (*P* = 0.01). There was no correlation between HER2 status of primary tumors (PT) and *HER2*-status of CTC-enriched blood fractions (P = 0.87) (**Table 5**).

**Table 4:** Results of univariate and multivariate logistic regression analysis in relation to lymph node involvement.

Gene expression status	Number of cases (%)		Univariate analysis		Multivariate analysis	
	N-	N+	OR (95% CI)	*P*	OR (95% CI)	*P*
***CK19***						
Negative	39 (80)	33 (67)	1.9 (0.75–4.8)	0.17		
Positive	10 (20)	16 (33)				
***MGB1***						
Negative	40 (85)	31 (66)	2.9 (1.06–8.2)	0.03	3.2 (1.1–9.2)	0.029
Positive	7 (15)	16 (34)				
***HER2***						
Negative	32 (67)	29 (62)	1.24 (0.5–2.9)	0.6		
Positive	16 (33)	18 (38)				
***VIM***						
Negative	43 (90)	32 (70)	3.76 (1.2–11.7)	0.02	4.2 (1.3–13.5)	0.01
Positive	5 (10)	14 (30)				
***TWIST1***						
Negative	47 (98)	47 (98)	1 (0.6–17)	1		
Positive	1 (2)	1 (2)				
***SNAIL***						
Negative	47 (98)	41 (85)	8.02 (0.92–69.9)	0.06		NS
Positive	1 (2)	7 (15)				
***CXCR4***						
Negative	32 (65)	25 (52)	1.73 (0.76–3.9)	0.19		
Positive	17 (35)	23 (48)				
***uPAR***						
Negative	25 (52)	15 (31)	2.46 (1.06–5.7)	0.03		NS
Positive	23 (48)	34 (69)				
***CK19/MGB1/HER2***						
Negative	29 (59)	15 (31)	3.29 (1.41–7.64)	0.005	Not applicable	
Positive	20 (41)	34 (69)				

N- lymph node involvement absent, N+ lymph node involvement present. OR-overall risk; CI – confidence interval, NS-not statistically significant.

**Table 5:** Correlation between CTC-enriched blood fractions gene expression status and clinicopathological parameters.

	*TWIST1*			*SNAIL*			*CK19*			*MGB1*			*VIM*			*CXCR4*			*uPAR*			*HER2*			CTC-EBF phenotype*		
Clinical variable	Neg.	Pos.	*P*	Neg.	Pos.	*P*	Neg.	Pos.	*P*	Neg.	Pos.	*P*	Neg.	Pos.	*P*	Neg.	Pos.	*P*	Neg.	Pos.	*P*	Neg.	Pos.	*P*	*CK19-/VIM+* **	*CK19+/VIM-* **	*P*
**T**																											
**T1**	41	1	1	39	3	1	29	14	0.25	35	5	0.02	38	3	0.009	31	12	0.01	21	21	0.10	29	13	0.34	1	7	0.04
**T2-4**	52	1		48	5		42	12		35	18		37	15		25	28		18	36		31	21		11	9	
**N**																											
**N-**	47	1	1	47	1	0.059	39	10	0.17	40	7	0.03	43	5	0.02	32	17	0.19	25	23	0.03	32	16	0.61	3	6	0.69
**N+**	47	1		37	7		33	16		31	16		32	14		25	23		15	34		29	18		9	10	
**Number of lymph nodes involved**																											
**≤3**	78	2	1	74	6	0.62	59	23	0.55	60	18	0.53	67	11	0.003	52	29	0.01	37	44	0.045	52	27	0.46	6	15	0.008
**>3**	16	0		14	2		13	3		11	5		8	8		5	11		3	13		9	7		6	1	
**Grade**																											
**G1-2**	65	1	0.53	62	4	0.25	49	18	0.91	48	16	0.86	52	11	0.34	40	26	0.59	30	36	0.22	41	24	0.73	7	13	0.23
**G3**	29	1		26	4		23	8		23	7		23	8		17	14		10	21		20	10		5	3	
**HR status**																											
**Negative**	14	0	1	13	1	1	10	5	0.53	11	3	1	13	2	0.73	8	7	0.64	8	7	0.30	12	2	0.07	1	1	1
**Positive**	80	2		75	7		62	21		60	20		62	17		49	33		32	50		49	32		11	15	
**HER2 status**																											
**Negative**	70	2	1	66	6	1	52	21	0.58	53	17	0.96	56	14	1	43	30	0.72	30	42	0.66	46	26	0.87	9	14	0.62
**Positive**	21	0		19	2		17	5		16	5		18	4		12	10		8	14		13	8		3	2	

Number of cases classified as positive (Pos.) or negative (Neg.) for a particular marker in each group are shown. Statistically significant *P* values are given in bold.

*CTC-enriched blood fraction phenotype – only *MBG1*+ and/or *HER2*+ fractions were considered

** *CK19−/VIM+* - mesenchymal phenotype; *CK19+/VIM−* - epithelial phenotype.

Multivariate analysis revealed that independent predictors of lymph nodes involvement were positive status of *MGB1* – OR 3.2 (95% CI 1.1–9.2, *P* = 0.029) and *VIM* – OR 4.2 (95% CI 1.3–13.5, *P* = 0.01) (**Table 4**).

**Figure 3:**
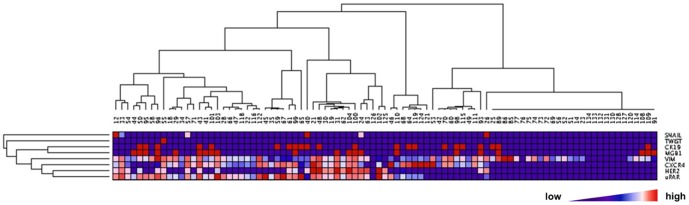
Unsupervised hierarchical clustering of relative gene expression values in CTC-enriched blood fraction. Samples containing missing values were excluded from the analysis. On the left-hand side cluster of CTC-enriched blood samples with elevated *VIM* (*P* = 0.068), *HER2, CXCR4* and *uPAR* (P<0.00001, for all three) expression in which lymph node involvement was more frequently observed (*P* = 0.04).

In hierarchical clustering, the study population was divided into two main groups, which differed in expression of *VIM, CXCR4, uPAR, HER2* (**Figure 3**). Patients in the cluster with elevated expression of these genes showed more frequent lymph node involvement (58%) than patients from the cluster with lower expression (35%; *P* = 0.03).

## Discussion

Dissemination of cancer cells is an early event and disseminated tumor cells can be found in bone marrow of patients with carcinoma in situ [46]. Nevertheless, in metastatic breast cancer more CTCs are seen than in early breast cancer patients [13], [16], [47]. No clear association is apparent between lymph node involvement and CTC detection rate. Some studies show similar CTCs detection rate in N− and N+ breast cancer patients [23], [48], which would indicate similar tumor seeding potential, but dissimilar colonization potential for disseminated cells. However, preliminary results from a large SUCCESS trial revealed a correlation between CTC presence and lymph node involvement [49], implying different seeding, and possibly colonization, potentials in N− and N+ patients. To explore differences in seeding and colonization potential we analyzed expression of mammary epithelial transcripts (*CK19, MGB1*), EMT-related factors (*TWIST1, SNAIL, SLUG, VIM*) and invasion and metastasis-related genes (*HER2, CXCR4, uPAR*) in CTC-enriched blood fractions from N- and N+ breast cancer patients. Considering the unresolved discussion concerning markers for CTC detection and characterization [50] and drawbacks of CTC-positive selection, we decided to apply a negative selection method for CTC-enrichment. Similar to prior research identifying CTCs with PCR-based methods, we used a “classical” definition of CTCs presence, meaning expression of either *CK19, MGB1* or *HER2* [23]. However, growing body of evidence shows that mesenchymal CTCs are not a rare event in cancers, what made us apply an extended definition of CTCs phenotypes – epithelial or mesenchymal – which includes *VIM* as an additional marker related to mesenchymal state. As a result we consider CTCs-enriched blood fractions being *CK19+/VIM−* and *MGB1+* or *HER2+* as containing CTCs in epithelial state, and *CK19−/VIM+* and *MGB1+* or *HER2+* fractions as carrying mesenchymal CTCs. Even though the phrase describing *MGB1* as “mammary epithelial transcript” implies it is present in mammary epithelium, it does not mean that it is expressed only by cells in the epithelial state. Although MGB1 function is not known, structurally related proteins are a group of secretory proteins binding steroid ligands that might present anti inflammatory activity [51], [52]; thus implying related function in human breast tissue, not restricted to epithelial state.

In our study, we found expression of either *CK19, MGB1* or *HER2* in 55% of the patients, which agrees with previous studies of early breast cancer patients [53], [54], [55]. Unlike in healthy controls, in DCIS cases expression of at least one CTC marker was detected, what supports results of the early cancer dissemination model [46]. We have observed significantly different CTCs detection rate (defined as expression of either *CK19, MGB1* or *HER2*) in N− and N+ patients (41% vs. 69%, respectively, *P* = 0.005; **Table 4**), which supports the notion that tumors from N+ patients have superior seeding potential compared with N− patients. We would not have drawn the same conclusion if *CK19* was the only CTC marker in our study, as in that case there would be no significant difference in CTC marker detection rate between N− and N+ patients (20% vs. 33%, *P* = 0.17; **Table 4**). Also, the study of Pecot indicates that cytokeratin is not an ultimate marker for CTC identification as cytokeratin-negative cancer cells can be found both in circulation and within PTs [56]. Multimarker approach for CTCs detection is therefore a necessity [14], [57]. Our immunofluorescence results also showed *CK19−/MGB1+* cells in CTC-enriched blood fractions, which was also seen in prior research [58]. Analysis of expression of EMT markers, and of invasion- and metastasis-related genes revealed that CTC-enriched blood fractions of N+ patients are more frequently *VIM*-positive and *uPAR*-positive (and show a trend toward being *SNAIL*-positive); and patients with more than three involved lymph nodes are often *VIM, CXCR4* and *uPAR*-positive. Increased metastatic abilities of tumor cells in N+ patients could be therefore attributed to expression of *CXCR4, uPAR* and *VIM* (and possibly *SNAIL*). *CXCR4* is a receptor for CXCL12 chemokine, which is secreted by the common sites of breast cancer metastasis, including lymph nodes [3]. Thus, *CXCR4* expression on CTCs could mediate homing of tumor cells to lymph nodes. Also, the role of *uPAR* in extracellular matrix degradation complex, migration and invasion could explain its metastasis-promoting function [34]. Amplification and increased expression of *uPAR* was observed before in CTCs [36], PTs, and disseminating tumor cells in bone marrow and in lymph nodes of breast cancer patients [59]. Co-expression of *uPAR* and *HER2* shown by Meng et al. [36] also supports our observations in *HER2*-positive CTC-enriched blood fractions, which were all *uPAR*-positive.

Although induction and maintenance of EMT are thought to require *TWIST1, SNAIL* and *SLUG*, their expression was not found in CTCs expressing mesenchymal markers [2], [60], nor were they highly expressed in our CTC-enriched blood fractions; only 8% and 2% of samples were positive for either *SNAIL* or *TWIST1*, respectively and *SLUG* was not detected in any sample. Similar rates were detected by Mego et al., who analyzed CTC-enriched blood fractions depleted of EpCAM-positive CTCs [22]. Despite the small number of *SNAIL*-positive samples, *SNAIL* expression correlated with positive status of *VIM*, *CXCR4, uPAR* and *HER2*. We also saw positive *VIM* status in 20% of the CTC-enriched blood fractions, which correlated with *SNAIL, CXCR4* and *HER2* expression. This result supports data showing increased malignancy of CTCs with mesenchymal phenotype, possibly resulting from EMT [20], [33], [61]. No significant increase in expression of *CXCR4* and *uPAR* in *CK19+/VIM−* CTC-enriched blood fraction would speak for the passive model of dissemination, in which tumor cells are physically translocated into the vasculature (or the neovasculature formed around tumor cells) [62]. In that case, cells would not need a migratory phenotype, typically associated with EMT, and could remain in their epithelial phenotype, which according to several theories would strengthen their metastatic ability because of increased adhesive properties. We presume that CTCs with mesenchymal phenotype (lacking epithelial adhesion molecules) acquire additional malignant properties (expression of *CXCR4* and *uPAR*) that allow successful lymph node colonization. It would be interesting to check whether expression of *CXCR4* and *uPAR* are related to any specific metastatic pattern other than the lymph node metastasis seen in our study.

Limitations of our study include relatively small sample size and short follow-up period, which hamper survival analysis. Moreover, the associations between variables are deduced from their inter-correlation, which does not necessarily inform about causal relationship. Therefore, our results should be seen as hypothesis-generating discoveries, and would obtain additional strength when supported by research aiming at deciphering molecular mechanisms behind them.

## Conclusions

In summary, our results show increased CTCs detection rate as well as more frequent *VIM, SNAIL*, and *uPAR*–positive rates of CTC-enriched blood fraction in patients with lymph nodes metastases. This indicates differences in both seeding potential and increased ability of the tumor cells of N+ patients to reach and divide in lymph nodes. We have also shown that *VIM*-positive (*CK19−/VIM+*) CTC-enriched blood fractions, unlike *CK19+/VIM−* fractions, show elevated *CXCR4* and *uPAR* levels, which might contribute to more aggressive tumor characteristics.

## Supporting Information

Figure S1 **Exemplary photos of immunostained cells isolated from CTC-enriched blood fractions of breast cancer patients.**
(PDF)

Figure S2 **Relative genes expression levels in breast cancer patients and controls.** Expression of *VIM, HER2, CXCR4, uPAR, TWIST1, SNAIL, CK19* and *MGB1* in CTCs-enriched blood fractions of breast cancer patients with invasive carcinoma (BC), patients with ductal carcinoma *in situ* (DCIS) and healthy controls (HC). As no sample expressed SLUG, results for this gene are not presented.
(PDF)

Table S1 **Spearman's rank correlations coefficients of relative gene expression levels in CTC-enriched blood fractions.**
(PDF)
