# Supplementary material for: Mesenchymal Phenotype of CTC-Enriched Blood Fraction and Lymph Node Metastasis Formation Potential
Source: PLoS One. 2014 Apr 7;9(4):e93901. doi: 10.1371/journal.pone.0093901 (PMC3977989; doi:10.1371/journal.pone.0093901)
Supplement: Figure S1 — Exemplary photos of immunostained cells isolated from CTC-enriched blood fractions of breast cancer patients. (PDF) [file pone.0093901.s001.pdf]

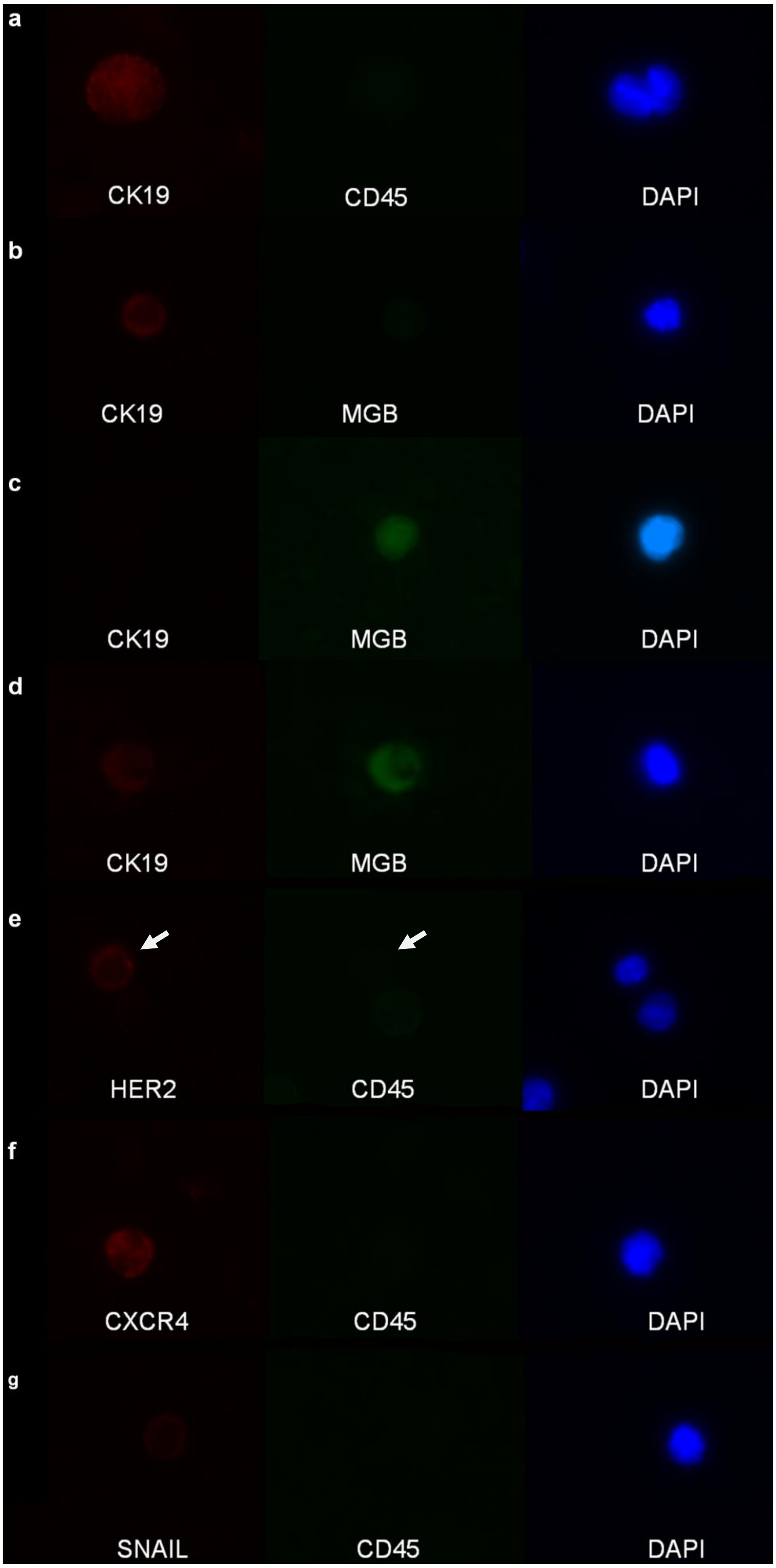

Figure S1. Exemplary photos of immunostained cells in CTC-enriched blood fractions of breast cancer patients. Each panel (a-g) represents results of double immunostaining with appropriated antibodies and DAPI. Following immunophenotypes are presented a) CK19+/CD45- b) CK19+/MGB1- c) CK19-/MGB1+ d) CK19+/MGB1+ e) HER2+/CD45- (marked with an arrow) f) CXCR4+/CD45- g) SNAIL+/CD45-.
