# Supplementary material for: Mesenchymal Phenotype of CTC-Enriched Blood Fraction and Lymph Node Metastasis Formation Potential
Source: PLoS One. 2014 Apr 7;9(4):e93901. doi: 10.1371/journal.pone.0093901 (PMC3977989; doi:10.1371/journal.pone.0093901)
Supplement: Figure S2 — Relative genes expression levels in breast cancer patients and controls. Expression of VIM, HER2, CXCR4, uPAR, TWIST1, SNAIL, CK19 and MGB1 in CTCs-enriched blood fractions of breast cancer patients with invasive carcinoma (BC), patients with ductal carcinoma in situ (DCIS) and healthy controls (HC). As no sample expressed SLUG, results for this gene are not presented. (PDF) [file pone.0093901.s002.pdf]

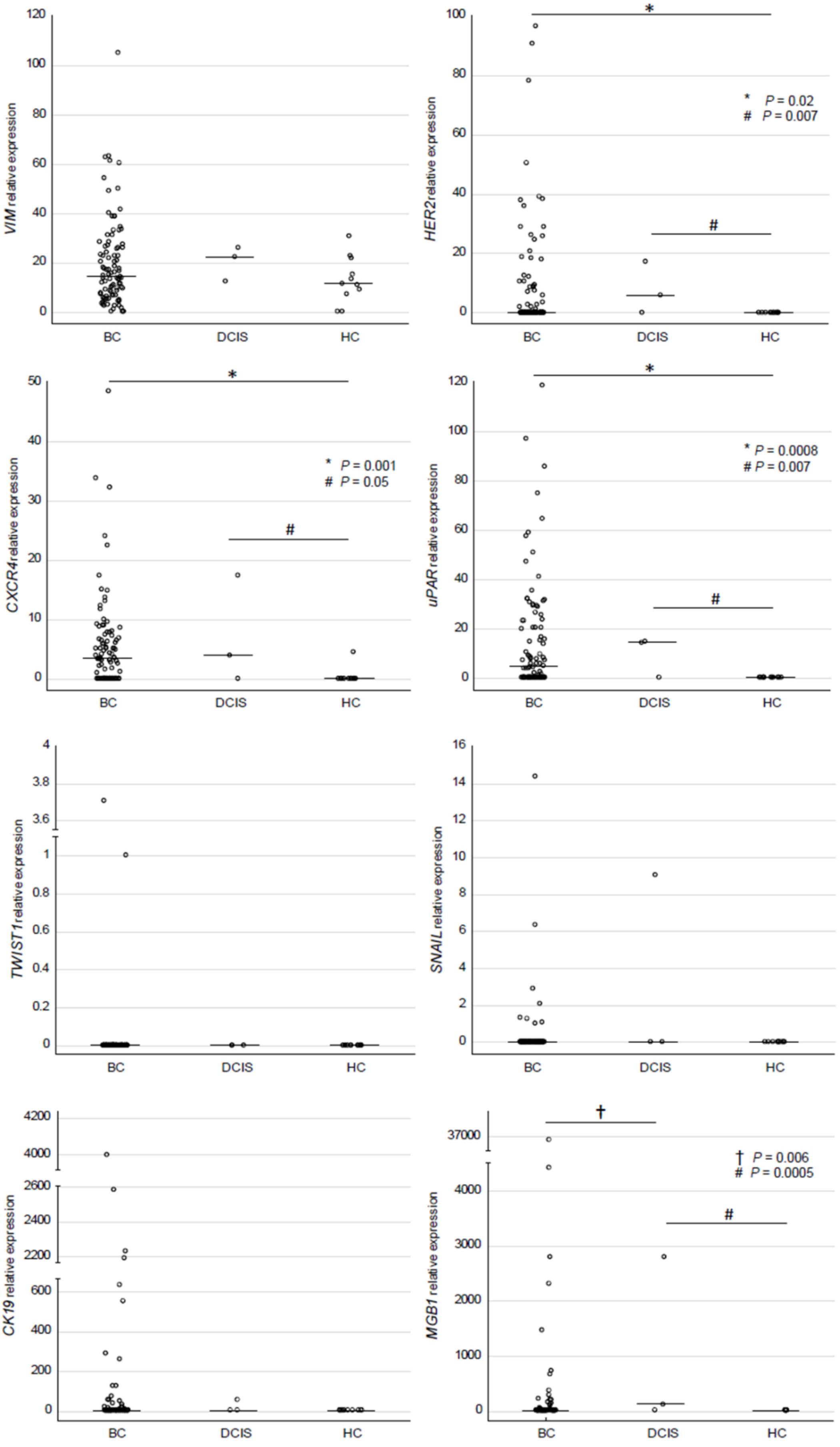

o relative gene expression level

— median expression level

† statistical difference between BC and DCIS

* statistical difference between BC and HC

# statistical difference between DCIS and HC

Figure S2. Relative genes expression levels in breast cancer patients and controls. Expression of *VIM, HER2, CXCR4, uPAR, TWIST1, SNAIL, CK19* and *MGB1* in CTCs-enriched blood fractions of breast cancer patients with invasive carcinoma (BC), patients with ductal carcinoma *in situ* (DCIS) and healthy controls (HC). As no sample expressed SLUG, results for this gene are not presented.
