# Supplementary material for: Mesenchymal Phenotype of CTC-Enriched Blood Fraction and Lymph Node Metastasis Formation Potential
Source: PLoS One. 2014 Apr 7;9(4):e93901. doi: 10.1371/journal.pone.0093901 (PMC3977989; doi:10.1371/journal.pone.0093901)
Supplement: Table S1 — Spearman's rank correlations coefficients of relative gene expression levels in CTC-enriched blood fractions. (PDF) [file pone.0093901.s003.pdf]

Table S1. Spearman's rank correlations coefficients of relative gene expression levels in CTC-enriched blood fractions. Statistically significant values are given in bold.

	VIM	***TWIST1***	***SNAIL***	***MGB1***	***HER2***	***CXCR4***	***uPAR***
CK19	-0,217<br>**P=0.03**	-0,083<br>P=0.42	-0.108<br>P=0.29	0.542<br>**P<0.00001**	0.046<br>P=0.66	<0.001<br>P=1	0.021<br>P=0.84
	VIM	0.018<br>P=0.87	0.210<br>**P=0.04**	-0.134<br>P=0.20	0.283<br>**P=0.006**	0.580<br>**P<0.00001**	0.536<br>**P<0.00001**
		TWIST1	0.220<br>**P=0.03**	0.045<br>P=0.66	0.172<br>P=0.10	0.059<br>P=0.67	0.102<br>P=0.33
			SNAIL	-0.086<br>P=0.41	0.401<br>**P=0.00006**	0.353<br>**P=0.0004**	0.353<br>**P=0.0004**
				MGB1	0.065<br>P=0.54	0.022<br>P=0.84	0.113<br>P=0.28
					HER2	0.537<br>**P<0.00001**	0.649<br>**P<0.00001**
						CXCR4	0.766<br>**P<0.00001**
							uPAR
